# Curcumin in Retinal Diseases: A Comprehensive Review from Bench to Bedside

**DOI:** 10.3390/ijms23073557

**Published:** 2022-03-24

**Authors:** Davide Allegrini, Raffaele Raimondi, Alfredo Borgia, Tania Sorrentino, Giovanni Montesano, Panos Tsoutsanis, Giuseppe Cancian, Yash Verma, Francesco Paolo De Rosa, Mario R. Romano

**Affiliations:** 1 Eye Center, Humanitas Gavazzeni-Castelli, 24128 Bergamo, Italy; davideallegrini@yahoo.it (D.A.); tpanos02@gmail.com (P.T.); mario.romano.md@gmail.com (M.R.R.); 2 Department of Biomedical Sciences, Humanitas University, 20100 Milano, Italy; alfrborgia@gmail.com (A.B.); tania.sorrentino@humanitas.it (T.S.); giuseppe.cancian@gmail.com (G.C.); yash.verma@st.hunimed.eu (Y.V.); derosafrancescopaolo@gmail.com (F.P.D.R.); 3 Optometry and Visual Sciences Department, University of London, London WC1E 7HU, UK; giovmontesano@gmail.com

**Keywords:** curcumin, review, carrier, diabetic retinopathy, retinitis pigmentosa, macular degeneration

## Abstract

Recent evidence in basic science is leading to a growing interest in the possible role of curcumin in treating retinal diseases. Curcumin has been demonstrated to be able to modulate gene transcription and reduce ganglion cell apoptosis, downgrade VEGF, modulate glucose levels and decrease vascular dysfunction. So far, the use of curcumin has been limited by poor bioavailability; to overcome this issue, different types of carriers have been used. Multiple recent studies disclosed the efficacy of using curcumin in treating different retinal conditions. The aim of this review is to comprehensively review and discuss the role of curcumin in retinal diseases from bench to bedside.

## 1. Introduction

Photoreceptors and retinal ganglion cells (RGCs) are susceptible to oxidative stress, resulting in the generation of reactive oxygen intermediates (ROIs). ROIs are produced by aging, irradiation, inflammation, air pollution, cigarette smoke and other factors. Singlet oxygen, free radicals and hydrogen peroxide are all included in ROIs. Superoxide anions, hydroxyl free radicals, hydroperoxyl radicals and lipid peroxyl radicals are types of free radicals. All of these factors contribute to oxidative stress, which disrupts the anti-oxidant system, resulting in DNA fragmentation, proteolysis, lipid fragmentation, protein cross-linking, apoptosis and cell necrosis. The retinal pigment epithelium (RPE) and photoreceptors both possess anti-oxidant enzymes [1]. The fact that the retina consumes much more oxygen than any other tissue makes it vulnerable to oxidative stress and, as a result, generates ROIs.

A variety of natural products, including carotenoids (zeaxanthin and lutein), gingko biloba, saffron, catechin have been proven to have significant neuro-protective effects and the capability to normalize the histological and functional alterations in animal models [2,3,4,5].

Curcumin is a powerful antioxidant that has been used for a thousand years in traditional Chinese medicine and is now widely diffused worldwide with pleiotropic applications [6].

Turmeric is a plant of Asian origin belonging to the Zingiberaceae family (the same family as ginger). The species of greatest interest is Curcuma longa, which is cultivated in most tropical regions, particularly in India. In order to grow, the plant needs a temperature between 20 and 30 °C and a fair amount of annual rainfall. 

The Curcuma longa plant consists of an underground root (rhizome) from which, once it is dried and ground, a powder with a characteristic yellow-orange color can be obtained that is rich in various active ingredients with numerous biological activities [7]:

- Curcuminoids (95% of the standardized extract): curcumin, demethoxicurcumin and bisdemethoxycurcumin
- Volatile oils: tumerone, atlantone and zingiberene

The most abundant element, and also the most studied, is curcumin, which, like the other curcuminoids, belongs to the polyphenol family. Curcumin makes up 2 to 8% of the weight of common turmeric powder for cooking. Curcumins are composed of two phenolic groups linked by two unsaturated α, β groups. The latter, as acceptors, favor nucleophilic additions.

Clinical research carried out in recent years confirmed the remarkable anticancer, anti-inflammatory and antioxidant properties of curcumin. Furthermore, a potential neuro-protective effect of curcumin itself has recently been demonstrated. The safety of curcumin, when taken at a low dose, is supported by significant evidence, including evidence from various human clinical studies as well as a variety of experimental animal and in vitro studies that further corroborated the human observations. Clinical studies have shown hepatotoxicity at high doses [8].

The anti-inflammatory property of curcumin is caused by a blockage of TNF-dependent NF-kB (tumor necrosis factor dependent transcriptional nuclear factor kappa B) and paths that generate reactive oxygen intermediates [7].

The use of curcumin in age-related macular degeneration was investigated on cellular models, demonstrating a decreased apoptosis of retinal pigmented epithelial cells and a reduction of inflammatory markers [9,10]. So far, the use of curcumin has been limited by poor bioavailability; to overcome this issue, different types of carriers have been used [11]. Starting from recent evidence in basic science, there is a growing interest in the possible role of curcumin in treating retinal diseases. The aim of this review is to provide an updated and comprehensive overview from bench to bedside of curcumin’s safety and effectiveness for treating retinal diseases.

## 2. Literature Search

A comprehensive search was performed by using Embase, MEDLINE, Web of Science and Google Scholar to ensure efficient coverage [12]. The search was performed in September 2021 and updated in March 2022. Used terms were “curcumin and retina”, “macular degeneration and curcumin”, “retinitis pigmentosa and curcumin”, “retinal disease curcuma”, “diabetic retinopathy and curcumin” and “curcumin human trials”, yielding 337 results. Abstracts were reviewed and relevant articles were selected. From these articles, cited publications were also selected if related to the topic of this review.

## 3. Molecular Mechanisms

### 3.1. Gene Transcription to Reduce Ganglion Cell Apoptosis

Several studies have shown mechanisms for gene transcription to reduce retinal ganglion cell apoptosis. In particular, Lu et al. showed how curcumin is able to block the growth of retinal murine ganglion cells (N18) in vitro (the growth inhibition effect) through the induction of apoptosis mediated by a down-regulation of the B-cell lymphoma 2 gene (Bcl-2) and the up-regulation of the protein BAX [13]. Curcumin is also able to interact in the mechanisms of apoptosis through the up-regulation of some active forms of caspases, such as the caspases 3, 8 and 9, and through inhibiting the expression of genes involved in DNA repair, such as 6-methylguanine-DNA methyltransferase (MGMT), DNA-dependent protein kinase (DNA-PK), breast cancer gene 1 (BRCA1), 14-3-3r, ataxia telangiectasia and the Rad3-related protein (ATR) and ATM serine/threonine kinase (ATM) [13,14,15].

**Figure 1:**
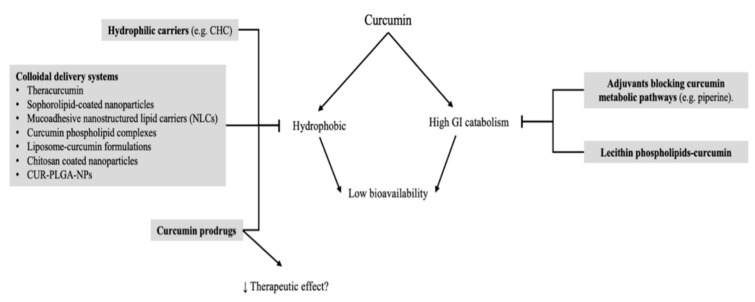
Schematic representation of molecular mechanisms involved retinal ganglion cell transcription and vascular endothelial growth factor levels. Down arrow stands for reduction.

Other studies performed in vitro have shown that curcumin, when administered in a low dosage, is able to reduce SS-mediated cell death; however, it is toxic at a high dosage. Nonetheless, the administration of 10 μM of curcumin in vivo is able to mitigate protease-mediated retinal ganglion cell (RGC) and amacrine cell death significantly. Furthermore, curcumin shows a protective effect by restoring nuclear factor kappa-light-chain-enhancer of activated B cells (NF-kB) [16] (Figure 1).

### 3.2. Vascular Endothelial Growth Factor Reduction

The vascular endothelial growth factor (VEGF) is implicated in several retinal conditions, such as diabetic retinopathy, AMD, premature retinopathy and retinal vascular occlusions [17,18,19,20]. There are at least five cell types capable of producing VEGF, including retinal pigment epithelium cells; macroglia cells, such as astrocytes and Muller cells; ganglion cells and vascular endothelial cells [21,22,23,24]. Hypoxia inducible factor 1 (HIF-1) has a key role in the expression of VEGF under hypoxic conditions [25]. Studies have discussed how curcumin is able to reduce VEGF production through the regulation of HIF-1 [26,27]. Premanand et al. have shown how curcumin may induce endothelial retinal human-cell apoptosis in vitro [28]. Concurrently, Kowluru et al. described how curcumin administration is able to reduce serum VEGF levels in diabetic rats [29]. Mrudula et al. studied the effects of curcumin on VEGF expressions in the streptozotocin-induced diabetic rat retina (STZ), demonstrating that curcumin inhibits the expression of VEGF in the retina under hyperglycemic conditions [30] (Figure 1). Moreover, curcumin is implicated as an aryl hydrocarbon receptor (AhR) activator [31,32]. The AHR signaling pathway has a crosstalk with different major cell-signaling pathways, including a crosstalk with HIF-1. AHR knockout mice do not exhibit traits of low vision that interfere with successful development; however, these mice do show alterations in vascular structures in the eye [33]. Indeed, AHR has a crucial role in the vascular development of the eye.

### 3.3. Glucose Level

Several studies on rodent models of diabetes have found that the oral administration of various dosages of curcumin is able to reduce levels of glucose [34] and of glycosylated hemoglobin (HbA1C) in blood [35] and to improve insulin sensitivity [36,37].

Dietary curcumin (0.5% in a diet) has also been seen to be effective in ameliorating increased levels of fasting blood glucose, urine sugar and urine volume in streptozocin (STZ) induced diabetic rats [38] (Figure 1).

The possible mechanisms of the effect of curcumin on glycemia may be explained by different molecular mechanisms of action.

The activity of curcumin in the control of hyperglycemia is expressed mainly by down-regulating α-glucosidase and α-amylase activity and activating enzymes in the liver associated with glycolysis, gluconeogenic and lipid metabolic process [39].

Curcumin is also beneficial for insulin-producing and insulin-responsive tissues, such as liver, skeletal muscle and adipose tissues [40]; in fact, the levels of protein kinase B (Akt) phosphorylation and glucose transporter type 4 (GLUT4) translocation in skeletal muscles are increased by curcumin. Moreover, curcumin can elevate plasma insulin levels and increase lipoprotein lipase (LPL) activity [39].

Curcumin has the ability to activate peroxisome proliferator-activated receptor- gamma (PPAR-𝛾) in rat hepatic stellate cells [41]. It is known that PPAR-γ agonists, such as troglitazone, pioglitazone and rosiglitazone, are able to stimulate adipocyte differentiation, thus having hypoglycemic effects.

There is an improvement in pancreatic islets in diabetic mice treated with curcumin for 12 weeks; curcumin seems to reduce lymphocyte infiltrations and to lead to an increase in the number of small islets of Langerhans near the ducts in the pancreas [42].

Curcumin can decrease the levels of thiobarbituric acid reactive substances (TBARS) and the activity of sorbitol dehydrogenase (SDH) [35,43,44] that are higher in diabetic rats and contribute to vascular dysfunction and protein glycosylation. In fact, an increased flux through the polyol pathway reduces the effectiveness of the glutathione redox cycle in scavenging free radicals, and it is known that the free radicals, if not scavenged, damage the vascular endothelium. However, SDH catalyzes the conversion of sorbitol to fructose in the presence of NAD, leading to an increased availability of fructose, and that fructose is a 10-fold better substrate than glucose for glycosylation.

**Figure 2:**
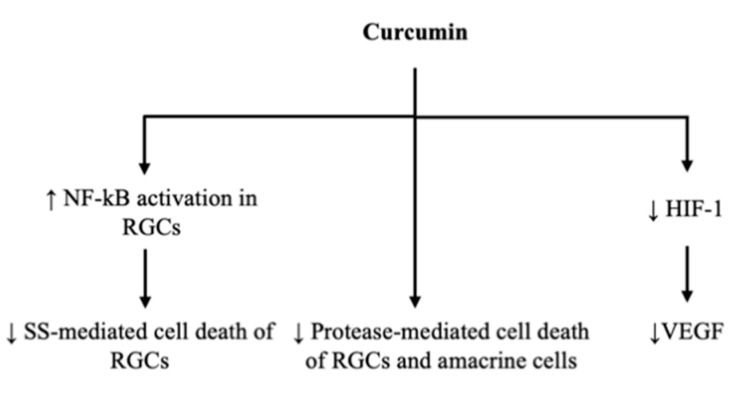
Schematic representation of molecular mechanisms leading to curcumin-mediated reductions in glucose levels. Up arrow stands for augmentation, down arrow stands for reduction.

Finally, curcumin reduces the levels of tumor necrosis factor-α (TNF-α) [45] and plasma-free fatty acids (FFA) that correlate with insulin resistance. It also inhibits nuclear factor-kappa B (NF-κB) activation, [46] protein carbonyl, lipid peroxidation [45] and lysosomal enzyme activities (N-acetyl-β-d-glucosaminidase, β-d-glucuronidase and β-d-galactosidase), that are good intra-individual indicators of the metabolic control of the disease [38] (Figure 2).

### 3.4. Vascular Dysfunction

In the early stage of diabetes, there is an increase in pro-inflammatory proteins, such as intercellular adhesion molecule-1 (ICAM-1), inducible nitric oxide synthase (iNOS) and vascular endothelial growth factor (VEGF), that leads to an increased leukocyte adhesion to retinal vessels and a consequent blood–retinal barrier (BRB) breakdown [47,48,49].

This process is modulated by calcium/calmodulin-dependent protein kinase II (CaMKII), which is a protein kinase that regulates transcriptional activity on NF-kB and the consequent release of inflammatory cytokines [50,51,52].

Activated CaMKII plays an important role in the development of abnormal vascular dysfunction in diabetes, including diabetic retinopathy; in fact, autocamtide-2-related inhibitory peptide, a potent inhibitor of CaMKII, prevents vascular leakage in diabetic mice [53,54,55].

Curcumin significantly inhibits the activation of CaMKII/NF-κB signaling induced by diabetes or elevated glucose levels, and it subsequently decreases the expression of VEGF, iNOS and ICAM-1. These changes are associated with a decrease of diabetes-induced retinal vascular leakage [56] (Figure 1).

## 4. Pharmacokinetics

Curcumin is a hydrophobic polyphenol derived from the rhizome of the herb Curcuma longa. Regardless of the presence of studies regarding its utility and safety, its efficacy is limited by its poor bioavailability in humans, even when it is orally administered in high doses (12 g/day). Several factors are responsible for this. For example, curcumin is retained at the pre-enterocytic level by gastrointestinal mucus, and the latter contains enzymes able to modify curcumin, thus leading to a pre-enterocytic biotransformation. Curcumin’s bioavailability is further worsened by its interaction with enterocytic proteins, which modify its structure [57].

Another important contributor to curcumin’s unfavorable pharmacokinetic profile is the fact that absorbed curcumin undergoes extensive phase I (modification) and phase II (conjugation) metabolisms, generating mainly curcumin glucuronide and curcumin sulfate [58], mostly at the levels of the liver, the intestine and gut microbiota. Curcumin’s double bonds are subsequently reduced in enterocytes and hepatocytes by a reductase to dihydrocurcumin, tetrahydrocurcumin, hexahydrocurcumin and octahydrocurcumin [59,60]. However, several in vitro studies demonstrated that curcumin’s metabolites themselves possess significant antioxidant activities, thus possibly playing a major therapeutic role [60].

Several studies demonstrated the poor bioavailability of curcumin.

A study [61] evaluated the pharmacokinetics of a single high dose of oral curcumin and its ability to induce heme oxygenase 1 (HO-1) expressions among humans with different specific (GT)n polymorphisms. HO-1 acts as a protective enzyme through anti-inflammatory, anti-oxidant, anti-apoptotic and anti-proliferative mechanisms. A longer (GT)n repeat polymorphism in the promoter region of the HO-1 gene corresponds to a lower transcriptional activity of the HO-1 promoter region. Moreover, curcumin strongly up-regulates HO-1 activity. Five subjects with a homozygous short (S/S) GT genotype and five with a homozygous long (L/L) GT genotype were enrolled in an open-label uncontrolled phase-one pilot study. They were administered 12 g of oral curcumin after each conducted an overnight fast. Their curcumin and total bilirubin plasma levels, HO-1 mRNA and protein expressions in their peripheral-blood mononuclear cells (PBMCs) were analyzed before the study and at 2.5, 5, 7.5, 10, 24 and 48 h. The study showed that despite a single high oral dose of curcumin, no measurable curcumin plasma levels could be detected, nor could any changes in the HO-1 expressions. Moreover, no significant difference was encountered in the two groups.

Another study [58] assessed the pharmacokinetic profile of curcumin and its conjugated metabolites in healthy humans administered through the arm in either a 10 g or a 12 g single dose, the highest doses given to humans. Free curcumin was detected in the plasma of only one subject. However, a rapid increase in curcumin conjugates was detected in plasma, thus showing how curcumin may induce its own biotransformation in the gut epithelium or the liver. Interestingly, the Cmax and area under the curve (AUC) data for the curcumin conjugates in the 10 g arm doses were significantly higher than those in the 12 g arm doses. This may be explained by the saturation of a transport mechanism resulting in no incremental curcumin absorptions and the induction of curcumin metabolisms at higher doses. Moreover, two subjects reported loose yellow stools the day after their curcumin administration, most likely due to poor curcumin absorption.

Curcumin’s poor bioavailability encouraged scholars to find various solutions to the issue. Among these, Theracurmin, a new form of curcumin, should be listed. A phase one study was performed, aimed at investigating the safety and the pharmacokinetics of curcumin in 16 pancreatic or biliary tract cancer patients who failed standard chemotherapy [62]. Daily oral Theracurmin was added to gemcitabine-based chemotherapy. Ten patients were administered curcumin doses of 200 mg, and six were administered doses of 400 mg. The study demonstrated that Theracurmin can safely increase plasma curcumin levels in a dose-dependent manner and that its bioavailability cumulatively increases after its repeated administration. Other options aimed at increasing curcumin’s bioavailability investigated adjuvants blocking the metabolic pathways of curcumin as piperine, an inhibitor of hepatic and intestinal glucuronidation. Nanoparticles, liposomes, micelles and phospholipid complexes are other promising novel formulations [57]. A study [63] was conducted on 50 healthy humans to determine the safety and the pharmacokinetics of a liposomal formulation of curcumin developed for intravenous administrations. Different doses were investigated (10, 20, 40, 80, 120, 180, 240, 320 and 400 mg/m^2^). The highest dosage group reported adverse reactions, including red blood-cell mean corpuscular volume (MCV) increases and echinocyte formations. Such morphological changes were transient and asymptomatic; therefore, the close monitoring of subjects may be enough to prevent dangerous events, such as hemolysis. The intravenous administration of liposomal curcumin resulted in a dose-dependent increase in plasma curcumin and its metabolite tetrahydrocurcumin (THC). After the termination of the infusions, the plasma levels of both curcumin and THC dropped rapidly. The observation that plasma exposure to THC post-infusion does not decrease as much as for curcumin suggested that curcumin cleared rapidly from the plasma into tissues is still being metabolized into THC post-infusion. Another study [64] compared the pharmacokinetic profile of a novel natural dried-turmeric colloidal suspension with four other turmeric formulations at their recommended doses, including a standardized extract, a combination-containing piperine, a phytosome formulation and a liquid micellar preparation. Thirty healthy men and women were enrolled in this randomized open-labelled crossover trial. The micellar preparation delivered higher levels of total curcuminoids than any other formulation. The total curcuminoid absorption levels were also significantly higher for the dried colloidal suspension when compared with either piperine or phytosome formulations, which, surprisingly, did not demonstrate improvements in curcuminoid bioavailability.

## 5. Carriers and Bioavailability

As previously mentioned, curcumin was found to be poorly soluble in water; the maximum solubility of it in an aqueous buffer (pH 5.0) was reported to be as low as 11 ng/mL [65]. The limited solubility of curcumin, its extensive systemic metabolism and its rapid degradation when it is in contact with GI fluids are responsible for the low bioavailability of curcumin after its oral delivery. In addition, curcumin in a solution may be sensitive to UV light, so a marked photochemical degradation could occur under UV exposure, leading to a difficulty in its handling for clinical use [65]. To increase the bioavailability of curcumin, many formulations have been developed that greatly increase it while keeping the dosages needed to acquire desired concentrations in blood flow. Many researchers have been using AUC as the most reliable measurement of the biological availability because it takes into account the entire response over time, whereas *C*max measures only one point in time and is therefore less robust [66].

**Figure 3:**
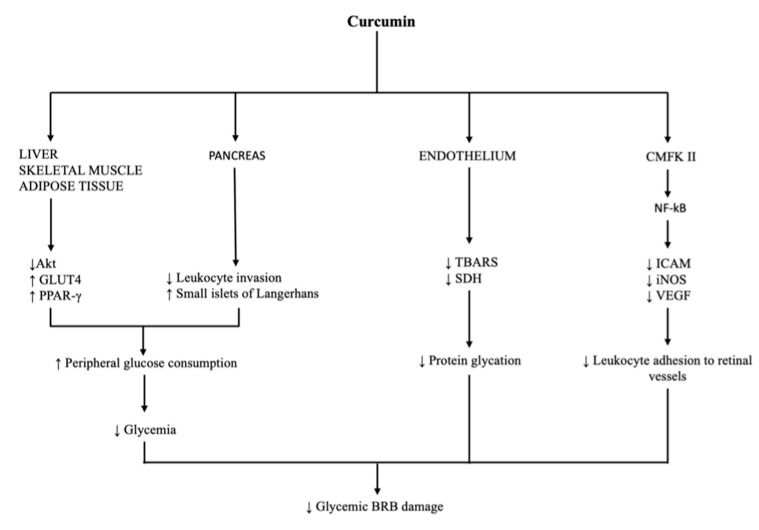
Diagram reviewing options to enhance curcumin delivery. Up arrow stands for augmentation, down arrow stands for reduction.

Theracurmin is a formulation proven to increase the bioavailability of curcumin 16- to 27-fold (as measured by using AUC) in both humans as well as rats compared to curcumin powder [65,67]. Another effective formulation is a combination of curcumin with a hydrophilic carrier, cellulosic derivatives and natural antioxidants (CHC), which shows a 45.9-fold higher absorption compared to unformulated curcumin in healthy volunteers [68]. Furthermore, adjuvants, which can block the metabolic pathways of curcumin, are being used to improve its bioavailability. Piperine is an inhibitor of hepatic and intestinal glucuronidation that was combined with curcumin and showed an increase of 157% in the bioavailability in rats and a 2000% increase in the bioavailability in humans compared to curcumin alone [57]. Research has been ongoing regarding the encapsulation of curcumin within colloidal delivery systems that contain small particles dispersed within an aqueous medium, such as emulsions, nanoemulsions, micelles, liposomes, biopolymer microgels and polymer nanoparticles. Biosurfactants are used to increase the stabilities of the particles in these colloidal delivery systems. Sophorolipids are surface-active glycolipids composed of a polar sophorose group and a nonpolar fatty acid group, which can be produced by using microbial fermentation and can be used as biosurfactants. Sophorolipid-coated nanoparticles were used in both in vitro and in vivo studies, which showed that curcumin nanoparticles have an appreciably higher bioavailability than free curcumin crystals (2.7–3.6-fold), which is mainly attributed to their higher bioaccessibility [69]. Mucoadhesive nanostructured lipid carriers (NLCs) loaded with curcumin showed a greater (by 2-fold) in vitro adhesion to porcine intestinal mucosa compared to curcumin powder, which prolongs the contact time of the drug delivery system, thus improving the therapeutic efficacy of curcumin [70]. Moreover, NLCs made up of curcumin mixed with taurocholic acid as a ligand showed a 5–15-fold increase in curcumin’s bioavailability [71]. Additionally, studies on nanoemulsions used as delivery systems for curcumin have been conducted, all showing increased bioavailabilities [72,73,74]. The phospholipid complex can increase the bioavailability and absorption of curcumin due to the amphiphilic nature of complex, which increases curcumin’s water and lipid solubility. Through different studies, a 1.5–5-fold increase in curcumin’s half-life was found for the curcumin phospholipid complex over free curcumin [11]. Liposomes can be described as phospholipid bilayers surrounding an aqueous core and have been used in formulations with curcumin. Studies by Chen et al. [75] and Makoto et al. [76] showed an enhanced bioavailability with liposome–curcumin formulations. Ongoing research on curcumin prodrug developments is being done where promoieties are attached to the phenolic hydroxyl groups via a biodegradable linkage; their biological evaluation has mainly been limited to in vitro toxicity, and a possible lack of bioactivation in an in vitro model should be considered [77]. A formulation made up of a lecithin phospholipid–curcumin (Meriva^®^) complex increases the bioavailability over 20 times compared to plain curcumin; it does so by placing curcumin in a phytosome made of phospholipids in order to protect it from intestinal hydrolysis [7]. As mentioned above, curcumin has a poor water solubility; therefore, attempts to combine it with chitosan, a biodegradable and biocompatible polymer that is relatively water soluble, have been made. Chitosan and curcumin can be crosslinked into nanoparticles or synthesized into chitosan-coated nanoparticles, and using chitosan-based magnetic nanoparticles can be used where a magnetic field permits the precise targeting of the drug to a specific location in the body [78]. Other compounds that are being investigated are the poly(lactic-co-glycolic acid) nanoparticles (PLGA-NPs) of curcumin (CUR), named CUR-PLGA-NPs, which are thought to be associated with an improved water solubility, a higher release rate in intestinal juice, an enhanced absorption due to an improved permeability, the inhibition of P-glycoprotein (P-gp)-mediated efflux and an increased residence time in the intestinal cavity. When tested, CUR-PLGA-NPs showed a 5.6-fold bioavailability raise and a longer half-life when compared with native curcumin [79]. Finally, a study conducted by Schiborr et al. showed that a micronized curcumin formulation had 14-, 5- and 9-fold better bioavailabilities while a micellar curcumin formulation had 277-, 114- and 185-fold better bioavailabilities than native curcumin in women, men and all other subjects, respectively. Therefore, apart from presenting that these formulations of curcumin gave much better bioavailabilities than native curcumin, it also revealed sex differences with respect to the plasma AUC of curcumin. Women absorbed curcumin to a larger extent more (higher Cmax and AUC) than men [80] (Figure 3).

## 6. Safety Profile in Humans

Cheng et al. investigated the pharmacokinetics, toxicities and therapeutically effective dosages of curcumin in patients with premalignant or high-risk lesions. In 25 patients, a 500 mg dose was given daily, then the dose was escalated to 1000, 2000, 4000, 8000 and 12,000 mg tablets; in doses up to 8000 mg, no toxicity was seen. However, the patients did not like the 12,000 mg dose due to the tablet’s bulkiness. The serum peak was detected 1–2 h after the ingestion, and it steadily decreased over the next 24 h, with minimally noticeable serum levels up to 2000 mg and undetectable urine excretions. When the curcumin was administered for three months, it had no toxicity up to 8000 mg. Despite the fact that one patient with CIN and leucoplakia developed frank malignancies, some of the patients’ histological conditions improved [81] Lao et al. conducted a study with 24 suitable participants who had not received curcumin-rich food in the previous 14 days to determine the maximum tolerated dose and safety of curcumin. Its safety was tested for 72 h after a dose escalation of 500–12,000 mg of standardized curcumin; according to the National Cancer Institute’s common toxicity criteria (version 2.0), there were seven grade-one adverse events: headaches, rashes, yellow stools and diarrhea. There was no toxicity linked to the dose. Only two participants demonstrated serum concentrations of curcumin at dosages of 10,000 and 12,000 mg; the low serum levels could be attributed to the hepatic and intestinal metabolic bio-transformations [82].

## 7. Evidence in Treatment of Retinal Diseases

Because of its several molecular effects that might be beneficial to retinal tissues, curcumin has been studied as a therapeutic agent for a wide array of retinal pathologies in the last 20 years.

### 7.1. Age-Related Macular Degeneration and Choroidal Neovascularization

Age-related macular degeneration (AMD) represents one of the principal causes of blindness in the aging population globally [83]. The initial phases of the disease are characterized by alterations of the retinal pigmented epithelium (RPE) in the parafoveal region, while the late phases are associated with geographical atrophy and choroidal neovascularization [84]. It is acknowledged that the degenerative conditions of RPE cells related to oxidative stress constitute a key role in the AMD pathogenesis [85]. In fact, following inadequate cell repair and regeneration, oxidative stress can cause an accumulation of abnormal lipids and proteins. Over the past 20 years, several studies have shown that antioxidant agents can improve age-related changes associated with oxidative damage in RPE cells. AREDS studies have discussed the ability to reduce oxidative stress through the use of vitamin-based supplements (vitamin C, vitamin E and beta-carotene), the integration of fatty acids, as well as the use of zinc and copper additives [86].

Curcumin is efficacious in treating age-related macular diseases in both in vitro and animal models. Several authors have demonstrated its ability to protect retinal pigmented epithelium cells from oxidative stress by acting as an ROS scavenger, as a miRNA modulator and as an inducer of antioxidant molecules in ARPE-19 cells (an in vitro model of RPE cells) [9,10,87].

The use of curcumin supplements as a therapeutic possibility in retinal pathologies associated with oxidative stress and inflammation has been described by several studies in the literature [4,5,6,7,8,9]. In particular, Mandal et al. showed that curcumin down-regulates nuclear factor-jB (NF-jB), which is crucial in cellular inflammation, resulting in the guaranteed structural and functional protection of retinal cells from light-induced retinal degeneration in mouse models [88]. Howell et al. described the effects of curcumin in the post-transcriptional regulation of identified microRNAs associated with anti-oxidant systems [86]. Zhu et al. described the effects of curcumin on the genetic regulation of factors involved in inflammation in AMD; in particular, the study showed that curcumin, by decreasing the expressions of genes for the oxidation biomarkers superoxide dismutase (SOD), glutathione and maleic dialdehyde, reduced the apoptosis rate in RPE-aged cells [9]. Other studies showed that curcumin could have an important role in the cellular-defense augmenting nuclear-related factor (Nrf2), an important cytoprotective protein, and in boosting heme oxygenase-1, an enzyme involved in AMD pathogenesis [10,89].

**Figure 4:**
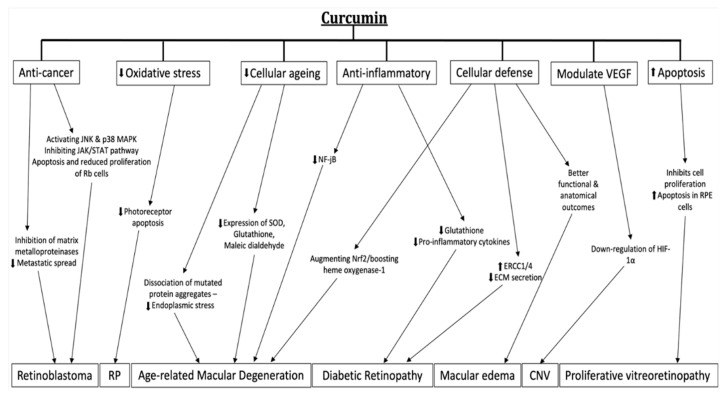
Schematic diagram reviewing curcumin mechanism of action in retinal diseases. Up arrow stands for augmentation, down arrow stands for reduction.

As previously discussed in the molecular mechanisms section, several studies have shown how curcumin can modulate the production of VEGF mediated by HIF-1 and NF-kB [26,27]. In addition, Xie et al. showed the therapeutic effects of curcumin on the down-regulation of HIF-1a in a mouse model with a laser-induced choroidal neo-vascularization (CNV) [90]. Therefore, curcumin may be used as a therapeutic adjuvant in cases of choroidal neo-vascularization. Allegrini et al. [91] demonstrated that in wet AMD patients receiving a curcumin-based supplement, the number of anti-VEGF injections that was required was significantly lower (Figure 4).

### 7.2. Diabetic Retinopathy

Almost all patients with diabetes mellitus for at least 20 years develop some signs of retinopathy. The structural anomalies of the retinal capillaries, on the basis of the pathophysiology of diabetic retinopathy, are as follows: a loss of pericytes, loss of endothelial cells, thickening of the capillary basement membrane and dysfunction of endothelial cells.

These alterations produce a loss of vascular self-regulation and of the barrier function, the uncontrolled diffusion of liquid and solutes from the capillaries into the extracellular space of retina sensorineural hearing loss and the narrowing of the capillary lumen [92].

In this context, curcumin seems to play an important role in the pathogenetic mechanisms of the disease through its anti-inflammatory, anti-angiogenic and hypoglycemic effects. In mice models, the oral administration of curcumin demonstrated a decrease in glutathione levels, a decrease in pro-inflammatory cytokines and an increase in antioxidant molecules in the retinal tissue [93]. Moreover, curcumin can reverse the pathological process that leads retinal cells affected by DR to secrete less extracellular matrices by increasing mammalian excision repair cross-complementing 1 (ERCC1) and ERCC4 levels [94]. Another target of curcumin in DR is the micro-vasculature; in fact, it is able to prevent the tortuosity, shrinkage, narrowing, microaneurysm formation and blind ending of retinal arteries, choriocapillaris and veins [95]. Another study managed to demonstrate that curcumin’s intake lowers the VEGF levels measurable in retinal tissues [30]. All the previous data were obtained from studies that tested curcumin on streptozotocin-induced diabetic rats; data on its effects on human eyes is still lacking (Figure 4).

### 7.3. Retinitis Pigmentosa

Retinitis pigmentosa is a slowly progressive and bilateral degeneration of the retina and retinal pigment epithelium caused by various genetic mutations. Its inheritance can be autosomal recessive, autosomal dominant or, rarely, X-linked. A beneficial effect of curcumin administrations was seen in this condition: Emoto et al. demonstrated that intravitreal injections of curcumin were able to reduce photoreceptor apoptosis, the main feature of the disease, in mice models [96]. Another study assessed the effects of curcumin in rats with the P23H mutation in the rhodopsin gene, one of the mutations responsible for RP. It was proven that curcumin induces a dissociation of mutated protein aggregation and diminishes endoplasmic reticulum stress; moreover, it improves the retinal structure, function, gene expression and localization of rhodopsin [97].

### 7.4. Macular Edema

In the central part of the retina, i.e., the macula, there is a delicate cellular balance that allows optimal nutrition in the cells responsible for vision with the maintenance of physiological conformations and therefore also with the maintenance of vision [98].

However, there are diseases with which this balance is lost slowly over time, such as in diabetic retinopathy or in an acute form, such as in thrombosis, inflammatory forms or after cataract surgery.

In such cases, the fluid that forms, which is called an edema, is not removed; it accumulates in the retina and vision worsens. Regarding this condition, Ferrara et al. [18] reported better functional and anatomical outcomes in patients with macular edemas from central serous chorioretinopathy and post-operative macular edema (Irvine-gass) receiving a curcumin-based supplement on a daily basis (Figure 4).

### 7.5. Proliferative Vitreoretinopathy

Proliferative vitreoretinopathy (PVR) is a common complication of retinal detachment surgery for which pathogenetic features are the migration, de-differentiation and proliferation of RPE cells. PVR is believed to be an abnormal wound-healing reaction that is primarily driven by inflammatory, retinal and RPE cells [99]. Currently, surgery is the only management option for PVR as there is no established pharmacological agent for the treatment or prevention of PVR. Several in vitro studies demonstrated that curcumin might be effective in countering these processes by inhibiting cell proliferation, inducing apoptosis and inducing cell necrosis in RPE cells [100,101,102] (Figure 4).

### 7.6. Retinoblastoma

Retinoblastoma occurs with a frequency of 1/15,000–1/30,000 live births and accounts for approximately 3% of childhood malignancies. It is typically diagnosed in children younger than five years old. Cancer can be hereditary; its heredity is mainly autosomal dominant but with an incomplete penetrance. About 25% of patients have a bilateral form, which is always hereditary. Another 15% of patients exhibit a unilateral hereditary form, and the remaining 60% exhibit a non-hereditary unilateral form. The pathogenesis of the heredity appears to involve the mutational deactivation of both alleles of a retinoblastoma tumor-suppressor gene (RB1) located on chromosome 13q14 [103]. In the inherited form, a mutation in the germline changes one allele in all cells, and subsequently, a somatic mutation alters the other allele in the retinal cells, which results in the tumor. The non-hereditary form probably implies a somatic mutation of both alleles in a retinal cell.

Lastly, in the literature, it was postulated that, thanks to the various effects of curcumin therapy described above, the substance could also have anti-cancer properties [104]. In vitro experiments on human Y79 retinoblastoma cells showed that curcumin seems to have anti-tumor effects in the treatment of retinoblastoma, the most common retinal cancer, by modulating the miRNA expression profile [105], activating the Janus kinase (JNK) and p38 MAPK pathways [106] and inhibiting of the JAK/STAT pathway, leading to a reduced proliferation, migration, invasion and increased apoptosis of Rb cells [107]. Another in vitro study focused on the effects of curcumin on mouse-rat hybrid retina ganglion cells (N18) and managed to demonstrate that the compound might be effective in preventing the metastatic spread of cancer cells through the inhibition of matrix metalloproteinases [107] (Figure 4).

## 8. Human Clinical Trials on Curcumin

While current clinical trials on retinal diseases are limited, many clinical trials have demonstrated curcumin’s therapeutic benefits in Alzheimer’s disease. In a six-month randomized placebo-controlled double-blind clinical trial [108] across varying dosages of curcumin, an increase in vitamin E levels was detected that positively correlated to the total curcuminoid levels. It was noted that serum Ab40 levels increased, thus proposing that curcumin may have the ability to disaggregate Ab deposits, promoting clearance. Clinical trials assessing the impacts of curcumin on insulin resistance showed a decrease in circulating GSK-3β and IAPP levels over 12 weeks [109]; this demonstrated a positive effect of glycemic control through a decrease in insulin-resistance fasting serum insulin.

Based on a human trial investigating the effects curcumin on blood lipid levels, the consumption of curcumin did not exhibit any significant alterations in serum lipid profiles [110]. Human trials investigating curcumin’s effects on cognitive function found there to be limited evidence for curcumin’s proposed benefit to improving cognitive function [111]. It should be noted that the duration of the study was 12 months, and therefore, it proposed a further investigation to focus on a longer intervention duration or an older age range (Table 1).

A recent human trial investigated the role that curcumin may play in reducing age-related increases in LV afterloads in addition to lifestyle modifications [112]. Researchers found a significant decrease in aortic systolic blood pressure, HR-corrected aortic AP and AIx in a group that combined curcumin and regular endurance exercise. It should be noted that this was not found in groups undergoing monotherapy with curcumin or regular endurance exercise. The study had a sample size of 45 post-menopausal women, and future studies should follow a larger sample size and different patient populations [112].

## 9. Conclusions and Perspectives

Curcumin has demonstrated promising results in treating retinal diseases; however, the evidence for as well as reproducibility of these are limited. Bioavailability is the main challenge; new carrier and formulations demonstrate good efficacies. Randomized controlled clinical trials are needed to establish, with a rigorous scientific approach, curcumin’s efficacy and safety in treating retinal diseases.

## Figures and Tables

**Table 1:** Table reviewing human clinical trials using curcumin.

Authors	Year of Publication	Sample (*n*=)	Duration	Main Findings
Baum et al. [108]	2008	34	6 months	Curcumin may have the ability to disaggregate Ab deposits
Thota et al. [109]	2020	29	12 weeks	Better glycemic control through a decrease in insulin-resistance fasting serum insulin
Baum et al. [110]	2007	36	6 months	No effect of curcumin on blood serum profiles
Rainey-Smith et al. [111]	2016	96	12 months	Curcumin treatment group had no cognitive decline at six months compared to placebo group
Sugarawa et al. [112]	2012	45	8 weeks	Higher decrease in aortic systolic blood pressure, HR-corrected aortic AP and AIx in the group taking curcumin and practicing regular endurance exercise

## Data Availability

Data available upon request.
